# Myocarditis associated with *Salmonella enterica* serovar Weltevreden gastroenteritis in medical practitioner; case report from South India

**DOI:** 10.3389/fmed.2025.1513974

**Published:** 2025-03-19

**Authors:** Mahadevaiah Neelambike Sumana, Morubagal Raghavendra Rao, Vidyavathi B. Chitharagi, Badveti Satyasai, Supreeta R. Shettar, Veerabhadra G. S. Swamy, Chinchana S. Eshwarappa, Yogeesh D. Maheshwarappa

**Affiliations:** Department of Microbiology, JSS Medical College and Hospital, JSS Academy of Higher Education and Research, Mysuru, India

**Keywords:** non-typhoidal *Salmonella*, *Salmonella enterica* serovar Weltevreden, myocarditis, acute gastroenteritis, ciprofloxacin sensitive NTS

## Abstract

*Salmonella enterica* serovar Weltevreden, a non-typhoidal serovar, has emerged as a significant foodborne pathogen, particularly in Southeast Asian countries. While it is commonly associated with gastroenteritis and foodborne outbreaks, it can also lead to invasive infections in immunocompromised adults and neonates. This case report presents a rare instance of myocarditis associated with *Salmonella* Weltevreden gastroenteritis in a 43-year-old healthy male physician from South India. The patient had a month-long history of intermittent fever, which worsened 2 days before admission, along with myalgia and headache. A day after the admission the patient developed diarrhea. Upon investigation, stool culture revealed *Salmonella enterica* serovar Weltevreden. Notably, the patient had a genetic predisposition to inflammatory bowel disease and reported recent use of nonsteroidal anti-inflammatory drugs, which may have increased his susceptibility to non-typhoidal *Salmonella* infection. The patient also developed myocarditis, making this the first reported case of Weltevreden-associated myocarditis in the region. Antimicrobial susceptibility testing indicated ciprofloxacin susceptibility, an increasingly rare finding, as most reported serovar Weltevreden cases exhibit ciprofloxacin resistance. The patient recovered following treatment with ciprofloxacin and was discharged with instructions for follow-up. This case highlights the need for heightened awareness of the potential for non-typhoidal *Salmonella* infections to cause systemic manifestations, even in individuals without major underlying comorbidities. Continuous monitoring of antimicrobial resistance patterns and strict food safety measures are essential to control outbreaks of these emerging pathogens.

## Introduction

The burden of foodborne diseases is substantial. Every year, nearly 1 in 10 people fall ill, and 33 million healthy life years are lost. Diarrhoeal diseases are the most common illnesses resulting from unsafe food, with 550 million people affected annually, including 220 million children under the age of 5 years (1). *Salmonella* is recognized as one of the four major global causes of diarrheal diseases (1). Worldwide, non-typhoidal *Salmonella* (NTS) is estimated to cause approximately 93 million enteric infections and 1,55,000 deaths annually. In addition, invasive non-typhoidal *Salmonella* (iNTS) is associated with a global burden of 3.4 million cases and 681,316 deaths each year, with nearly two-thirds (63.7%) of these cases occurring in children under the age of five (2).

The majority of invasive NTS infections occur in sub-Saharan Africa and Europe. In certain sub-Saharan African populations, the incidence can exceed 100 illnesses per 100,000 individuals annually, and in young children, this figure may rise to over 1,000 cases per 100,000 individuals per year. In Europe, cases are predominantly reported from Russia, Ukraine, and Estonia. In contrast, regions such as America and Southeast Asia have significantly lower rates of iNTS infections, at 23 and 21 cases per 100,000 population, respectively. The Middle East and North Africa collectively report a negligible incidence of only 0.8 cases per 100,000 population. India is also notably impacted by NTS and iNTS infections. However, due to limited active surveillance, there is a lack of reliable data in the literature to accurately quantify the health burden associated with these infections (2, 3).

The global economic burden of non-typhoidal *Salmonella* (NTS) is substantial. According to a systematic review conducted by Sol Kim *et al.* in July 2024, the direct medical costs for both NTS and invasive NTS illnesses were estimated to range from USD 545.9 (Taiwan) to USD 21,179.8 (Türkiye) for NTS, and from USD 1973.1 (Taiwan) to USD 32,507.5 (United States of America) for invasive NTS per case. For NTS illnesses, the direct medical costs as a percentage of GDP per capita ranged from 0.3% (UK) to 198.4% (Türkiye). This significant variation highlights the differing economic impacts on patients across countries, with Türkiye experiencing a disproportionately high economic burden due to its lower GDP per capita and exceptionally high inflation rate in 2022. For invasive NTS illnesses, the direct medical costs as a percentage of GDP per capita ranged from 0.6% (US) to 42.6% (US). There were an estimated 53,5000 non-typhoidal *Salmonella* invasive disease illnesses and 77,500 deaths due to this disease in 2017 (1–3).

The genus *Salmonella* causes both Typhoidal and non-typhoidal salmonellosis, *Salmonella* Typhi, *Salmonella* Paratyphi A, and *Salmonella* Paratyphi B are responsible for Typhoidal Salmonellosis. Whereas a variety of other *Salmonella* serovars including *Salmonella enterica* serovar Weltevreden (*S.* Weltevreden) causes non-typhoidal salmonellosis (NTS) (3). Serovar Weltevreden has emerged as a dominant foodborne pathogen globally, particularly in Southeast Asian countries, over the past two decades. It is increasingly isolated from water, vegetables, meat, and seafood. *Salmonella* Weltevreden is associated with foodborne outbreaks in India, with reports from Assam, Kolkata, and Pune (4–6). In 2009, an outbreak of Serovar Weltevreden was reported from Mangalore, Karnataka, affecting 34 female students in a Nursing hostel (7). Serovar Weltevreden infection is not limited to gastroenteritis; it can also cause invasive infections such as sepsis, particularly in immunocompromised individuals. There are well-documented cases of serovar Weltevreden causing invasive infections both in our region (Karnataka, South India) and globally (8–10). Additionally, an intriguing case of *Salmonella* Weltevreden presenting as a lung abscess and empyema without preceding gastrointestinal symptoms has also been reported (10).

In this case report, we present a sensitive strain of *Salmonella enterica* serovar Weltevreden causing gastroenteritis and congestive gastropathy. However, several studies have reported multidrug-resistant serovar Weltevreden isolated from human and environmental sources. This serovar is also one of the most common organisms responsible for multiple outbreaks and invasive infections. Additionally, *Salmonella* Weltevreden is a complex serovar, with a more permeable genome than other *Salmonella* serovars. It also shows geographical clustering, although very few studies have reported this serovar in this region (Karnataka, South India). Therefore, reporting this case is particularly noteworthy.

## Case presentation

A 43-year-old male physician, presented to the medicine outpatient department with a history of intermittent fever over the past month, which had worsened in the last 2 days. He also complained of myalgia and headache. He had no known history of diabetes, hypertension, ischemic heart disease, or epilepsy, and there was no history of smoking, alcoholism, or drug abuse. The patient was admitted for the above complaints.

He was moderately built and well-nourished, general examination revealed a fever of 101^0^F with a pulse rate of 127 beats/minute, blood pressure was 140/90 mm Hg, and SPO_2_ showed a saturation of 91% in room air. The patient had no icterus, cyanosis, clubbing, lymphadenopathy, or edema but moderately raised jugular venous pressure and peripheral pulses were felt. The patient’s respiratory system examinations were normal. Hematological parameters revealed a total leucocyte count of 16,020 with neutrophil predominance (Neutrophilic leucocytosis). Upon admission, a provisional diagnosis of acute undifferentiated fever was made, and a blood sample was sent for culture and investigation of tropical fever causes, including enteric fever, dengue, leptospirosis, scrub typhus, and malaria.

Additionally, a cardiologist’s opinion was sought, and an Electrocardiogram (ECG) was performed, which showed flat T waves in lead I, aVL, Lead II, III, and aVF. T wave inversion was observed in V3-V6. Consequently, a 2D Echocardiogram was performed, which revealed normal valves and chambers with no regional wall motion abnormalities (RWMA). Left ventricular systolic function with an ejection fraction of 62% was noted. Based on these findings, an additional diagnosis of myocarditis was made, and the patient was started on an injection of doxycycline 100 mg BD (Bis in Die/Twice a day) to cover potential myocarditis caused by *Rickettsia*, which is a common etiology in tropical fever cases.

On the day of admission, the patient developed loose stools (4–5 episodes, with no blood and mucus). Consequently, a stool sample was sent for culture and microscopic examination. On the day following admission, loose stools continued (4–5 episodes), the patient also had a fever of 101.6°F and developed submandibular lymphadenopathy on the right side. Examination of the upper and lower respiratory tracts did not reveal any abnormalities. A chest X-ray showed blunting of the left costophrenic angle, and an ultrasound examination revealed bilateral minimal pleural effusion (approximately 30 ml), which was non-tappable. Additionally, a grade-I fatty liver and mildly oedematous ileal loops in the right iliac region were noted. With a history of diarrhea, an injection of ciprofloxacin 400 mg BD was started along with oral rehydration.

The serum samples sent for tropical fever panel analysis, including dengue NS1 and IgM antibodies, Weil–Felix, Widal, malarial lactate dehydrogenase antigen, and *Leptospira* IgM, all returned negative results. Blood culture showed no growth after 48 h of incubation. On the third day of admission, the patient also continued to have loose stools (4–5 episodes). His stool microscopic results revealed moderate inflammatory cells, no RBCs (Red blood cells), and no parasitic forms but stool culture revealed non-lactose fermenting colonies on MacConkey agar and green colonies on HEA (Hektoen enteric agar). Culture microscopy revealed motile, Gram-negative bacilli, which produced catalase but not cytochrome oxidase. The bacteria did not produce indole, urea was not hydrolyzed, but citrate was utilized. They reduced nitrate to nitrites and fermented glucose with gas production. The methyl red test was positive, but the Voges–Proskauer test was negative. The Triple Sugar Iron tube produced an alkaline slant and acid butt with abundant hydrogen sulfide, thus making differentiation between *Salmonella* Paratyphi B and *Salmonella* Typhimurium challenging. Hence, serotyping was performed using poly(O) antisera, HI, O2, O4, and O9 antisera and the isolate showed agglutination with poly(O) antisera and was negative for HI, O2, O4, and O9 antisera. Additionally, an automated identification system (Vitek-2 compact system version: 9.02, bioMérieux, France) identified the isolate as the *Salmonella* group.

The antimicrobial susceptibility testing (AST) was conducted using the Vitek-2 compact system version: 9.02 (bioMérieux, France) using an N-405 card, The version of CLSI used for MIC interpretation is a Copy of Global CLSI (2020) 30th edition. The isolate was sensitive to ampicillin, amoxicillin/clavulanic acid, ceftriaxone, trimethoprim/sulfamethoxazole, imipenem, meropenem, ciprofloxacin, and, chloramphenicol but was resistant to gentamicin, amikacin, and cefuroxime. The results of the Vitek-2 compact system were confirmed by the Kirby-Bauer disk-diffusion method. Quality control for both the Vitek-2 and disk-diffusion methods was routinely conducted following standard operating protocols guided by the M-100 CLSI (Clinical Laboratory Standard Institute) 30th edition. Additionally, the isolate was forwarded to the National Institute of Cholera and Enteric Diseases (NICED) in Kolkata, West Bengal, India, for speciation and serotyping. Currently, it is called as National institute for Research in Bacterial Infections (ICMR-NIRBI). The NICED/NIRBI report identified the isolate as *Salmonella enterica* serovar Weltevreden. The AST pattern along with minimum inhibitory concentrations, is shown in Table 1.

**TABLE 1:** Antimicrobial susceptibility pattern of *Salmonella enterica* serovar Weltevreden.

Antimicrobial agent	MIC (in μg/ml)	Interpretation
Ampicillin	≤2	Sensitive
Ceftriaxone	≤1	Sensitive
Imipenem	–	Sensitive
Meropenem	–	Sensitive
Chloramphenicol	–	Sensitive
Trimethoprim/ Sulfamethoxazole	≤20	Sensitive
Cefuroxime	–	Resistant
Gentamicin	–	Resistant
Amikacin	–	Resistant
Amoxicillin/Clavulanic acid	–	Sensitive
Ciprofloxacin	–	Sensitive

The antimicrobial susceptibility testing was performed using the Vitek 2 Compact system version 9.02 with the N-405 card. However, MIC values are not available for all antibiotics because, during cascade reporting, all antibiotics except ciprofloxacin, amoxicillin/clavulanic acid, trimethoprim/sulfamethoxazole, ampicillin, and ceftriaxone were blocked. The hospital information system report is available for these five antibiotics (attached). MIC values for other antibiotics are not included because the data was manually recorded in the laboratory’s culture logbook. We revisited the Vitek 2 system to retrieve data from the last year; unfortunately, the data had been erased. Additionally, we contacted BioMérieux technicians for assistance, but they also could not help us retrieve the data.

Once gastroenteritis due to NTS was diagnosed, the IV ciprofloxacin was discontinued, and initiated oral ciprofloxacin 500 mg BD for 2 days. By the fourth day after admission, the patient was afebrile but continued to experience loose stools (3–4 episodes per day, black in color). During the abdominal examination, mild hepatomegaly was noted, 1–2 fingers below the costal margin. High-resolution computed tomography (HR-CT) of the thorax revealed no additional findings apart from mild pleural effusion, and the patient was started on probiotics. The patient’s travel history was examined to identify the possible source of infection. He reported a positive travel history 2 weeks before hospital admission. His diet was non-vegetarian, and he and his family maintained proper personal and environmental hygiene. Psychosocially, the patient reported no stress or mental health issues, indicating a stable psychosocial environment. However, he did reveal a genetic predisposition to bowel disease within the family. But, the actual source of infection is not established.

On the 5th day of admission, the patient remained afebrile but passed semisolid stools with melena, 3–4 times a day. Consequently, an upper gastrointestinal endoscopy was planned for the following day. The patient reported a history of taking Diclofenac tablets continuously for 10 days before hospital admission. An antinuclear antibody test was performed to rule out autoimmune etiology, which came back negative. IV doxycycline was discontinued, and oral doxycycline (100 mg BD) was initiated because of myocarditis.

The next day, an upper gastrointestinal endoscopy was performed, revealing a lax lower oesophageal sphincter, normal fundus, congested mucosa of the body, and antral erosion. The impression from the endoscopy was congestive gastropathy. A biopsy taken during the procedure tested positive for the rapid urease test. Given the isolation of non-typhoidal *Salmonella* species in the stool, a diagnosis of congestive gastropathy with NTS was made. By this time, diarrhea had resolved, and the patient felt much better. The patient was discharged with anti-*Helicobacter pylori* treatment due to the positive result of the rapid urease test from the endoscopic biopsy specimen. He was advised to take oral doxycycline 100 mg BD for 14 days. The patient was also instructed to return to the hospital if warning signs such as fever, loose stools, haematochezia, or epigastric pain/burning occurred. Table 2 presents a timeline with relevant data from the episode of patients’ symptoms and treatment.

**TABLE 2:** Timeline with relevant data from the episode of patients’ symptoms and treatment.

Date	Case presentation	Treatment
Presentation to OPD/First day of admission	• Patient had a history of intermittent fever over the past month. • Fever worsened during last 2 days. • Patient complained of myalgia and headache • He had a fever of 101°F. • Hematology: Total leucocyte counts of 16,020 with neutrophil predominance. • Blood sample sent for Tropical fever analysis and blood culture. • ECG: Flat T waves in lead I, aVL, Lead II, III, and aVF. T wave inversion was observed in V3-V6. • Provisional diagnosis: AUF and Myocarditis. • On the day of admission, he developed loose stools (4–5 episodes, with no blood and mucus).	• The patient was started on IV doxycycline 100 mg BD
On the second day of admission	• Patient had a fever of 101.6°F. • He continued to have myalgia and headache. • He continued to have loose stools. • He developed submandibular lymphadenopathy on the right side. • Chest X-ray: Revealed blunting of the left costophrenic angle. • USG: Pleural effusion (approximately 30 ml). • Grade-I fatty liver. • A stool sample was sent for microscopic examination, culture, and AST.	• IV doxycycline 100 mg BD continued + IV ciprofloxacin 400 mg BD + Oral rehydration.
On the third day of admission	• Patient continued to have fever and loose stools. • Tropical fever analysis report returned negative. • Blood culture yielded no growth. • Stool microscopy: Moderate inflammatory cells, no RBCs, and no parasitic forms. • Stool culture: *Salmonella* Group. • Gastroenteritis due to NTS.	• IV doxycycline 100 mg BD continued + IV ciprofloxacin discontinued. • Initiated Oral ciprofloxacin 500 mg BD + Oral rehydration.
On the fourth day of admission	• AST report given in Table 1. • Patient continued to have loose stools. • Patient was afebrile. • Abdominal examination: Mild hepatomegaly,1–2 fingers below the costal margin. • HR-CT of thorax: No additional findings apart from mild pleural effusion.	• IV doxycycline 100 mg BD continued+ continued oral ciprofloxacin 500 mg BD + Oral rehydration.
On the fifth day of admission	• Patient was afebrile. • Passed semisolid stools with melena, 3–4 times a day.	• IV doxycycline discontinued • Initiated oral doxycycline (100 mg BD) • Continued oral ciprofloxacin 500 mg BD + Oral rehydration
On the sixth day of admission	• Upper gastrointestinal endoscopy: The impression from the endoscopy was congestive gastropathy. • Biopsy taken during the procedure tested positive for the rapid urease test. • Diagnosis of congestive gastropathy with NTS was made	• Continued oral doxycycline (100 mg BD) + continued oral ciprofloxacin 500 mg BD.
The next day, the patient was discharged with anti-*Helicobacter pylori* treatment. He was advised to take oral doxycycline 100 mg BD for 14 days. The patient was also instructed to return to the hospital if warning signs such as fever, loose stools, haematochezia, or epigastric pain/burning occurred.		

OPD, outpatient department; ECG, electrocardiogram; BD, Bis in Die/Twice a day; IV, intravenous; AUF, acute undifferentiated fever; USG, ultrasonography; AST, antimicrobial susceptibility testing; NTS, non-typhoidal *Salmonella*; HR-CT, high-resolution computed tomography.

The patient, being a medical doctor, shared the following about the treatment: As I had a fever on the day of admission with no localizing symptoms, Rickettsial infection was considered a possible cause of tropical fever. I was informed about this and started on IV Doxycycline 100 mg BD, which I felt was a rational treatment. The next day, after developing diarrhea with a moderate fever, I was started on ciprofloxacin 400 mg BD, with the role of ciprofloxacin in treating gastroenteritis explained to me. When the culture and sensitivity report arrived on day four, isolating the *Salmonella* group and its susceptibility to ciprofloxacin, I was switched to oral ciprofloxacin 500 mg BD for two additional days. I am happy to report that I improved with the remission of fever and diarrhea. I was also given an oral rehydration solution, which was rational due to diarrhea.

## Discussion

NTS infections significantly cause bacterial foodborne gastroenteritis and contribute to increased mortality in humans (2). These NTS are commonly found in animals, which acquire the bacteria through the consumption of raw produce contaminated by animal manure. The use of untreated animal manure as fertilizer can lead to soil and plant contamination with human pathogenic bacteria, posing a risk of pathogen transmission through fresh uncooked produce (11).

When the patient presents with acute undifferentiated fever, this case underscores the importance of cardiac evaluation. In this particular case, a previously healthy adult physician, who is 43 years old may not have any other reasons for myocarditis. After the completion of treatment, the patient being a physician himself got another ECG done that showed resolution of features of myocarditis. This implicates *Salmonella* Weltevreden infection as the cause of myocarditis. Myocarditis has been reported in infections with other serovars of *Salmonella* is detailed in the discussion.

Myocarditis associated with *Salmonella* Weltevreden is evident through ECG changes during the illness and the resolution of these changes following treatment. The most common cause of myocarditis is viral infections, which can include SARS-CoV-2, adenovirus (which causes the common cold), coxsackievirus (which causes hand, foot, and mouth disease), herpes virus, influenza (flu) virus, and parvovirus B19 (which causes fifth disease). Bacterial causes of myocarditis include *Staphylococcus, Streptococcus*, bacteria that cause diphtheria and Lyme disease. Fungal agents that can cause myocarditis include yeasts such as *Candida*, molds such as *Aspergillus*, and *Histoplasma* (often found in bird droppings). Parasitic causes of myocarditis include *Trypanosoma cruzi*, which causes Chagas disease, and *Toxoplasma*. The other causes were ruled out, as the patient did not exhibit any features suggestive of these conditions.

*Salmonella* Weltevreden is one of the most common non-typhoidal serovars associated with foodborne outbreaks in Southeast Asian countries and Europe, exhibiting geographical clustering in these regions. Notable outbreaks of serovar Weltevreden have been reported from Singapore in 1996; Malaysia from 1989 to 1994; Thailand from 1992 to 2002; Bangladesh in 2018; Norway, Denmark, and Finland in 2007; Réunion Island, a French Overseas Department, in August 2007; Northern Oman in 2015; and Southern and Coastal China in 2015 and 2016 (9, 11–15). The outbreaks were also reported in India. In 1996, a serovar Weltevreden outbreak was documented at a 650-bed teaching hospital in North India, persisting for over 9 months and affecting 84 healthy staff members and patients (16). Another outbreak occurred in Kolkata after an Iftar feast in North Dumdum in 2012. In Assam’s tea plantation, a serovar Weltevreden-associated foodborne gastroenteritis outbreak was reported, and in 2010, an outbreak affected students staying in a hostel in Pune, Maharashtra (4–6).

In our region (Karnataka, South India), a *Salmonella* Weltevreden gastroenteritis outbreak occurred in a ladies’ nursing hostel in Mangalore (Coastal Karnataka), involving 34 students. The infection was traced to a non-vegetarian dish provided by private caterers (7). Non-typhoidal *Salmonella* infections in humans typically begin with ingesting contaminated food or water. As a result, all outbreak reports emphasize ensuring food safety through strict food handling procedures and hygiene practices, including maintaining proper sanitation. Additionally, regular surveillance is strongly recommended to prevent future outbreaks.

In this report, we have isolated the serovar Weltevreden from a physician predisposed to inflammatory bowel disease, he reported a history of taking Diclofenac tablets continuously for 10 days because of fever. This predisposition, along with the use of nonsteroidal anti-inflammatory drugs, could have contributed to the patient’s susceptibility to acquiring NTS. Other key risk factors for acquiring *Salmonella* Weltevreden include a history of contact with infected animals, consumption of undercooked meat, poultry, eggs, and dairy products, ingesting contaminated seafood, and low stomach acidity (4, 7, 17).

We are reporting serovar Weltevreden-causing gastroenteritis in a 43-year-old male physician but Weltevreden infections are not limited to gastroenteritis, they can cause invasive infections in immunocompromised conditions including HIV, diabetes, malignancy, therapeutic immune suppression, co-infection with malaria, malnutrition, gastric hypoacidity, alteration of bowel flora, sickle cell disease, rheumatological disease, and extremes of age (5, 9, 17, 18). In this case, the patient’s extra-intestinal manifestation could have been due to an alteration in the gut flora as he is genetically susceptible to inflammatory bowel diseases.

In this case, *Salmonella* Weltevreden gastroenteritis is associated with myocarditis, marking the first reported case of Weltevreden-associated myocarditis in this region (India). Previously, a few studies have reported myocarditis caused by other NTS serovars. According to earlier reports, *Salmonella* Enteritidis is the most commonly implicated serovar responsible for myocarditis (19). NTS myocarditis is observed in both immunocompromised and immunocompetent adults and children. However, myocarditis in immunocompetent adults is rare. Per Sundbom *et al.* from Sweden reported a case of *Salmonella* Enteritidis myocarditis in a 22-year-old previously healthy male who developed chest pain following a period of profuse diarrhea, fever, and haematochezia. Magnetic resonance imaging confirmed the diagnosis of myocarditis, and stool culture was positive for *Salmonella* Enteritidis (20). Additionally, Lucy Childs and colleagues from Whipps Cross University Hospital, London, reported *Salmonella* Enteritidis-induced myocarditis in a 16-year-old girl. They emphasized the importance of considering *Salmonella*-induced myocarditis in patients with profuse NTS gastroenteritis, as these cases often present with non-specific clinical symptoms, making early diagnosis challenging (21). In addition to myocarditis, non-typhoidal *Salmonella* (NTS) serovars can also cause pericarditis. *Salmonella* Typhimurium is one of the common NTS serovars associated with pericarditis. In 1996, T. Bird reported a case of *Salmonella* Typhimurium pericarditis, and more recently, in 2023, F. Ladomenou *et al.* from Ioannina, Greece, and in 2024, N. Vigneswaran from Australia, also reported cases of *Salmonella* Typhimurium pericarditis (21–23).

Several studies have reported other invasive infections caused by *Salmonella* Weltevreden. In 2021, an unusual case of sepsis and respiratory distress syndrome with peritonitis in a 45-year-old male was reported by Janitha B. Gunasena *et, al* from Sri Lanka. Specifically, in our region (Karnataka, South India), invasive infections of serovar Weltevreden have also been reported. In 2004, two cases of invasive infections caused by serovar Weltevreden were documented. The first case involved a female baby weighing 2.7 kg, who developed umbilical sepsis and hypothermia, leading to septicemia. The second case involved a male neonate who developed hypothermia and septicemia. Both cases recovered successfully with treatment (8, 9). Additionally, in 2005, a case of post-cholecystectomy surgical site infection caused by *Salmonella* serovar Weltevreden was reported (7). Four cases of invasive infections due to *Salmonella* Weltevreden were also documented during an outbreak investigation in the USA in 2015 (24). Invasive infections caused by serovar Weltevreden are important to consider, given the emergence of this pathogen as a significant foodborne threat globally, particularly in Southeast Asian countries. Monitoring invasive infections is also crucial for tracking the rise of antibiotic resistance and ensuring the availability of effective treatment options for severe cases.

Antimicrobial resistance in serovar Weltevreden is minimal over time. The strain isolated in this case was sensitive to ampicillin, ceftriaxone, trimethoprim/sulfamethoxazole, ciprofloxacin, and chloramphenicol. Similar to this case a ciprofloxacin-sensitive serovar Weltevreden causing neonatal sepsis was reported in 2006 in this region (Karnataka, South India) (8). However, in recent years, it has developed resistance to various antibiotics. The genes responsible for antibiotic resistance in *Salmonella enterica* serovar Weltevreden are similar to those found in other members of the Enterobacteriaceae family. Several studies from India and globally have reported antibiotic resistance in *Salmonella* Weltevreden. Antibiotic resistance in *Salmonella* Weltevreden was observed during the outbreak linked to imported frozen raw tuna in the USA in 2015. Whole genome sequencing of *Salmonella* Weltevreden collected from human stool samples and contaminated food samples revealed that about eight isolates were resistant to tetracycline, ciprofloxacin, and ampicillin. The resistance was attributed to genes such as *qnrD*, *strA*/*strB*, *sul2*, *tetB*, and *bla*_*CTX–M–27*_ (15, 24).

In India, studies from Mangalore, Hubli, Assam, Maharashtra, and Rajasthan have reported multidrug resistance in *Salmonella* serovar Weltevreden isolated from human and animal samples. According to these studies, most *Salmonella* Weltevreden strains isolated from humans were resistant to ciprofloxacin, ampicillin, ceftriaxone, cephalexin, cefotaxime, cefoparazone, and cotrimoxazole. Isolates from animal and poultry samples showed resistance to ampicillin, tetracycline, cefotaxime, gentamicin, trimethoprim, ceftriaxone, chloramphenicol, ciprofloxacin, and cefepime, with resistance genes *bla*_*TEM*_, *tetA*, and *dfrA12* identified (4, 6–8, 12, 25).

Based on evidence, the patient was rationally treated with IV doxycycline to cover potential myocarditis caused by *Rickettsia* and IV ciprofloxacin for gastroenteritis. Subsequently, IV doxycycline was discontinued, and the patient was prescribed oral doxycycline for 14 days. These are key strengths of the case report. This case report presents a few key novelties in the context of existing literature. Myocarditis associated with Non-Typhoidal *Salmonella* (NTS) infections is rare, with *Salmonella* Enteritidis being the most commonly implicated serovar; however, this is the first documented case of myocarditis caused by *Salmonella* Weltevreden in an immunocompetent adult within our subcontinent. While invasive infections such as sepsis (umbilical sepsis), peritonitis, and respiratory distress syndrome caused by *Salmonella* Weltevreden have been reported in this region, there are no prior reports of myocarditis associated with *Salmonella* Weltevreden gastroenteritis. Additionally, the isolated serovar was ciprofloxacin-sensitive and also sensitive to ampicillin, ceftriaxone, and trimethoprim/sulfamethoxazole, in contrast to the increasing ciprofloxacin resistance observed in invasive NTS infections in recent years. Furthermore, this case underscores the importance of considering invasive infections in patients with a genetic predisposition to inflammatory bowel disease, even when caused by uncommon serovars such as *Salmonella* Weltevreden. Limitation of the study: the authors could not definitively establish the cause of myocarditis or pinpoint the exact source of the infection, which represents the report’s limitations.

## Conclusion

This case report presents rare myocarditis associated with *Salmonella enterica* serovar Weltevreden gastroenteritis in a previously healthy physician. *Salmonella* Weltevreden, commonly linked to foodborne outbreaks in Southeast Asia, highlights the potential for this serovar to cause significant illness even in individuals without major underlying comorbidities. The isolated serovar in this case, associated with myocarditis, marks the first reported instance of Weltevreden-associated myocarditis in this region (India). Additionally, the serovar exhibited susceptibility to multiple antibiotics, including ampicillin, ceftriaxone, and ciprofloxacin. This case underscores the importance of continuously monitoring antibiotic resistance patterns and emphasizes the need for enhanced food safety practices and surveillance systems to prevent outbreaks. Given the emerging nature of *Salmonella* Weltevreden in South India, further research and reporting of such cases are crucial for a better understanding of its clinical impact and epidemiological patterns in the region.

## Data Availability

The original contributions presented in this study are included in this article, further inquiries can be directed to the corresponding author.
